# The modified two-by-one fixed orthodontic appliance for bodily movement of canine: a case report

**DOI:** 10.1186/1757-1626-2-211

**Published:** 2009-11-19

**Authors:** Vicky WK Tsui, Hessa A Alkhal, Huie M Hou, Ricky WK Wong, A Bakr M Rabie

**Affiliations:** 1 Orthodontics, Faculty of Dentistry, the University of Hong Kong, 2/F, 34 Hospital Road, Sai Ying Pun, Hong Kong SAR, PR China; 2 Hamad Medical Corporation, Dental Department, P.O.Box 3050, Doha-Qatar; 3 24-1 Jalan Metro Perdana 8, Taman Usahawan Kepong, 52100 Kepong, Kuala Lumpur, Malaysia

## Abstract

**Background:**

Orthodontic management of an ectopically erupted canine requires substantial amount of bodily movement which is difficult to perform and often results in root resorption.

**Case presentation:**

A 9 year old girl presented with Class I malocclusion with crowding and ectopically positioned upper left canine (23). Treatment involved extraction of all first premolars. The alignment of the upper left canine was achieved by the modified 2 by 1 appliance.

**Conclusion:**

The design of this modified 2 by 1 appliance allows individualized bodily tooth movement of canine, it provides light and continuous force for physiological orthodontic movement with minimal root resorption.

## Introduction

The force magnitude, moment to force ratio, level of cellularity, vascularity and histomorphology of the supporting alveolar bone and the length and surface area of the root are critical factors affecting efficient tooth movement in orthodontics [1]. The unique morphology of the canine tooth coupled with its ectopic position of eruption makes control of root movement particularly difficult.

Orthodontic management of an ectopically erupted canine is very challenging. The condition usually require substantial amount of bodily movement of canine which is difficult to perform because the canine has a long and bulbous root. This morphology makes bodily movement of the canine time consuming, difficult to control and often results in root resorption.

Even when orthodontic forces are applied in a desired direction, it is difficult to produce the amount of root movement required because a large hyalinated layer will be created [1]. In addition, the canine root is usually close to the cortical bone of the maxilla, an area of reduced vascularization. This results in slow bone remodeling and tooth movement. In order to produce efficient canine root movement, very light orthodontic force will be needed.

We are introducing a modified 2 by 1 simple fixed orthodontic appliance to generate efficient directed tooth movement for the canine. It composed of several components as described below.

## 2 by 1 appliance

The appliance composed of the following components, for illustration; please refer to the figures in the following case report.

### Component one

Transpalatal bar This is stainless steel wire connecting the maxillary first molars. It is to preserve the posterior transverse arch width, stabilize posterior anchor teeth by minimising the mesial, buccal and rotational movements of the molars due to reaction force of other components of the appliance [2].

### Component two

0.016/0.018-inch Australian archwire on 0.022-inch slot preadjusted Edgewise bracket bonded on canine. The long span and the flexibility of the archwire reduce the load-deflection rate [3] and help deliver light and continuous force, provide the force necessary for physiological bone remodeling. Light and continuous force allows blood vessels to grow into the periodontal ligament; to bring in cells for bone remodeling; to minimize the hyaline layer formation and to minimize root resorption.

### Component three

U-loops mesial to molar buccal tubesThe U-loops are 3 mm in height stabilizing the arch wire and prevent sliding of the arch wire along the molar buccal tubes during orthodontic force application. Differential activation of the U-loops coupled with the canine stops, can provide directional control of tooth movements in three dimensions [4].

### Component four

Mesial and distal stops at canine bracketThis feature is a modification from the original 2 by 1 appliance. The stops are also constructed with U-loops but smaller than the molar U-loops, about 1.5 mm in height, they control the position of the canine and prevent sliding of wire along the canine bracket slot. A second-order band on the archwire inside the canine bracket creates a lace-back effect which provides an optimal force for root movement to occur. By activating the U-loops to press against the gingival area of the teeth they provide palatal root torque to the canine.

### Component five

Plastic tubings on the archwire between the molar and canine stops to prevent mucosa irritation of the archwire.

## Case Report

The following case report illustrates the use of this modified 2 by 1 appliance in the initial alignment stage of an ectopic maxillary canine. It produced root movement of the canine.

## History and clinical examination

A 9 year old Chinese girl was referred for the assessment of ectopically erupted upper canines. She had a convex lateral profile, acute nasolabial angle, protrusive lips and recessive mandible and chin.

**Figure 1:**
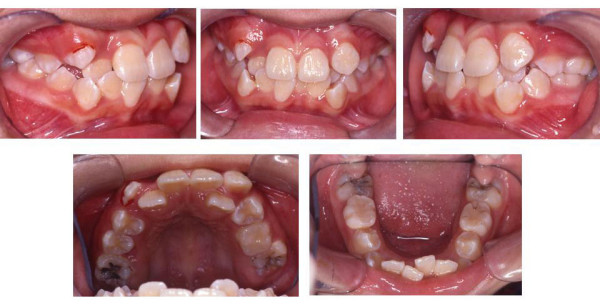
**Pre treatment intraoral view**.

Clinical examination (Fig. 1) showed severe crowding in upper arch, upper left canine (23) was ectopically erupted on the labial version of upper left lateral incisor (22), upper right (12) and left lateral incisors (22) were palatally displaced. There was 7 mm of crowding in the right maxilla and 9 mm of crowding in the left maxilla. Total of 5 mm crowding in the mandibular arch. Both the dental midlines were off to the left side by 1 mm. She had a Class II molar relationship with overjet of 1 mm and overbite of 1.5 mm. Functional mandibular displacement was noted on closing with premature contact at upper right lateral incisor (12) with lower right lateral incisor (42).

Radiographic examination showed a full permanent dentition, a mild Class II skeletal pattern and reduced lower facial proportion.

### Treatment plan and options

Due to the severity of crowding, the first option of treatment plan was to extract all the first premolars (14, 24, 34, 44) to provide space for alignment and correction of the dental midlines. Due to high anchorage demand and a large amount of root movement involved, an alternate treatment option was to extract both upper canines (13, 23) and lower first premolars (34, 44). The advantage of the latter option is the reduction in overall treatment time. The disadvantages are compromised aesthetic, difficulties to achieve good interproximal contact between the upper lateral incisors (12, 22) and the upper first premolars (14, 24) and a significant amount of recontouring of the palatal cusps of premolars might be needed. Thus the first option was adopted.

### Treatment progress

**Figure 2:**
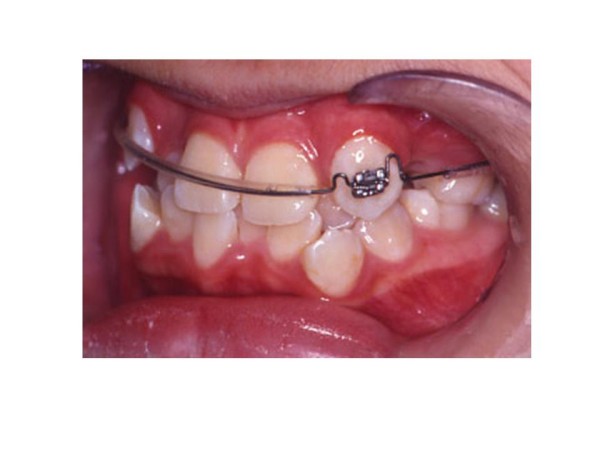
**The modified 2 by 1 appliance**: Left buccal segment-note the root of upper left canine (23) in mesial position.

**Figure 3:**
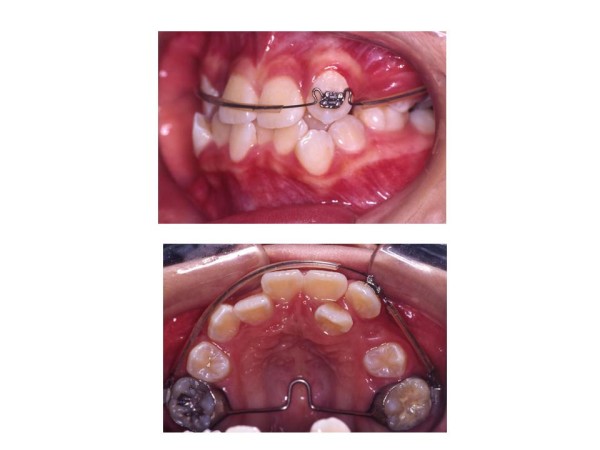
**Three months into treatment**. Figure illustrates the change of root position of upper left canine (23) to a distal direction (top), occlusal view (bottom).

The treatment began with extraction of upper first premolars (14, 24). A two by one appliance was constructed. Anchorage was enforced with a transpalatal arch bar, a pradjusted edgewise 0.022-inch slot bracket was bonded on upper left canine (23) only. 0.018-inch Australian stainless steel arch wire with U-loops mesial to upper first molars (16, 26) and mesial and distal to upper left canine (23) bracket was placed (Fig. 2). Differential activation of the arch wire thus delivered light and continuous force to tip the root of upper left canine (23) distally. (Fig. 3)

After 5 months of two by one appliance, the rest of the teeth in the upper arch, except the instanding upper lateral incisors (12, 22) were bonded for leveling and alignment with NiTi archwires. Upper left canine (23) was laced back to allow uprighting of the root.

**Figure 4:**
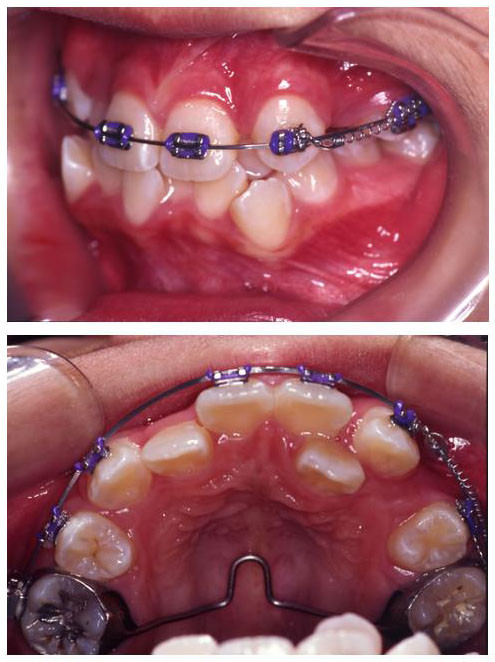
**Nine months into treatment**. Upper left canine (23) was retracted on 0.019 × 0.025-inch stainless steel wire with a nickel-titanium coil spring.

Retraction of upper left canine (23) on a 0.019 × 0.025-inch stainless steel arch wire was commenced in the ninth month of treatment (Fig. 4).

**Figure 5:**
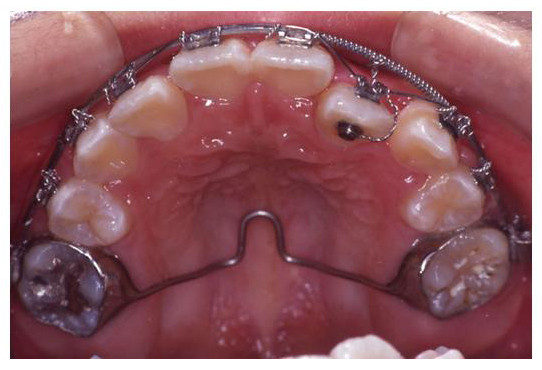
**Piggy back technique to align the in-standing**. The instanding upper left lateral incisor (22) was brought to the arch with piggy back technique.

The instanding upper left lateral incisor (22) was brought to the arch with piggy back technique (0.014-inch thermal NiTi archwire). Specifically, a segment of nickel-titanium wire is piggybacked onto a stainless steel wire in regions where flexibility is desired (Fig. 5).

Extraction of lower first premolars (34, 44) and placement of pre-adjusted appliance at the lower arch at the 18^th ^month treatment. To prevent dumping of the lower incisors, the teeth were only bond up after adequate space was created by canines retraction.

After arch coordination and finishing, the appliance was removed, retention involved upper and lower fixed retainer (0.018-inch Twistflex wire). The final records were taken 1 month after debonding.

## Results

**Figure 6:**
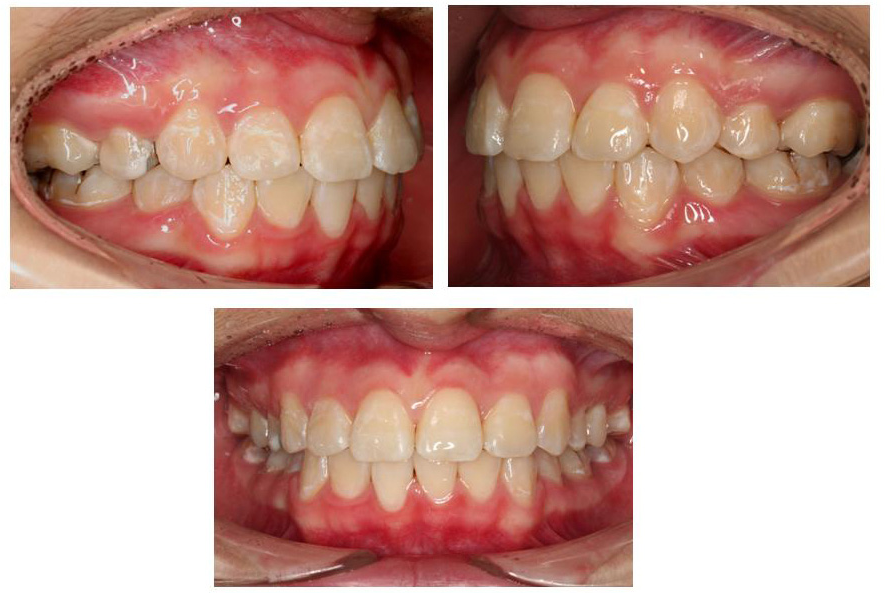
**Post treatment intraoral views**. Figure illustrates good occlusion and pleasing gingival contour.

Total treatment time was 48 month, this is partly related to the long treatment time required to totally retract the canines. Post treatment records show that the treatment objectives were achieved. Facial photographs show an improved profile and an attractive smile. Class I canine and molar relationships were established with canine-protected occlusion. Ideal overjet and overbite were achieved. Proper alignment and nice gingival contour were attained (Fig. 6). Despite regular appointments with dental hygienist and intensive oral hygiene instructions, oral hygiene of this patient still remained inadequate, demineralized spots were noticed.

**Figure 7:**
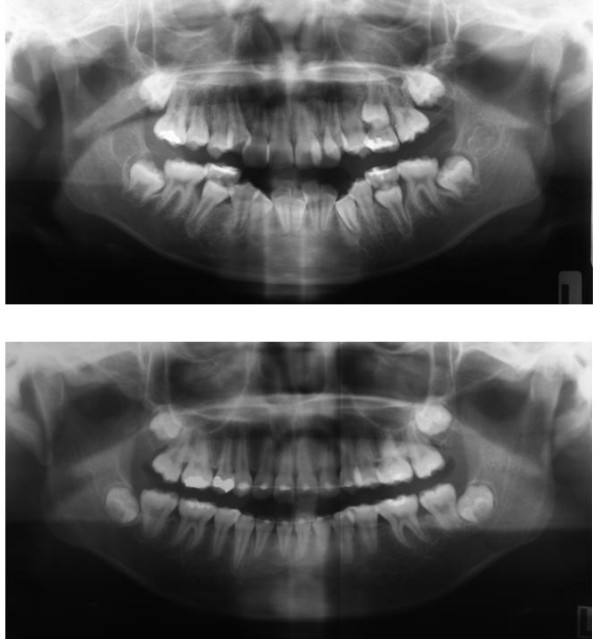
**Post treatment panoramic radiographs**. Post treatment panoramic radiograph showed reasonably root parallelism.

Post treatment panoramic radiographs show good parallelism of roots and normal structure of the periodontium. No sign of root resorption was seen (Fig. 7).

The post-treatment lateral cephalometric radiograph showed a balanced facial profile. Cephalometric analysis showed a Class I skeletal relationship, the ANB angle decreased slightly, as did the nasolabial angle. Dental measurements did not change significantly. A functional and good-looking occlusal result was achieved. The patient was satisfied with her teeth and profile.

## Discussion

The 48 months of treatment time may seem longer than average, but patients with ectopically positioned canines are perceived to be more time-consuming to treat than average orthodontic patients. It is of utmost importance in this case is to minimize undesirable side effects like the reactive moments on the anchor teeth, unwanted roots contacts and root resorptions. The anchorage on the transpalatal bar was possible to control the movement of the molars. With gentle, careful activation of archwire every visit, the amount of rolling of the round wire in the bracket or tubes will be negligible. Roots contact that may be caused by palatal root torque of canines can be avoided by carefully evaluating the proximity of the roots of the canine and the adjacent teeth clinically and with the radiographs. Despite the long treatment time, the root resorption in this case was minimal; this can only be achieved by careful, step by step orthodontic movement. The treatment time was well worth if we consider the biological result we achieved. It is also possible to using this appliance to deliver light force with torque control [5]. Further studies are needed to compare the effect of this appliance on orthodontic movement with other appliances e.g. using closing loops or nickel titanium coil springs.

## Conclusion

The design of this modified 2 by 1 appliance allows individualized bodily tooth movement of canine. The major components can provide light and continuous force for physiological orthodontic movement with minimal root resorption.

## Consent

Written informed consent was obtained from the patient for publication of this case report and accompanying images. A copy of the written consent is available for review by the Editor-in-Chief of this journal.

## Competing interests

The authors declare that they have no competing interests.

## Authors' contributions

RWKW designed the modified 2 by 1 appliance and supervised the treatment to the patient. VWKT was a major contributor in writing the manuscript. HAA, HHM and VWKT conducted the treatment. ABMR supervised the treatment to the patient.

## Authors' informations

Vicky W.K. Tsui BDS (HK), MOrth (HK); MOrthRCS (Edin), Private Practise in Hong Kong

Hessa A. Alkhal BDS (SA), MOrth (HK); MOrthRCS (Edin), MRACDS (Orth), Private Practise in Doha-Qata

Huie M. Hou BDS(Mal), MOrth(HK), MOrthRCS(Edin), Private Practise in Malaysia

Ricky W.K.Wong* BDS (HK), MOrth (HK), PhD (HK), FRACDS, MOrthRCS (Edin), FHKAM (Dental Surgery), FCDSHK (Orthodontics); Associate professor in Orthodontics

A Bakr M. Rabie BDS (Cairo), PhD (Northwestern), MSc (Northwestern), Cert Ortho (Northwestern), FHKAM (Dental Surgery), FCDSHK (Orthodontics), Hon FDSRCS (Edin), Professor in Orthodontics
